# UV-independent induction of beta defensin 3 in neonatal human skin explants

**DOI:** 10.12688/f1000research.5794.2

**Published:** 2015-02-19

**Authors:** Erin Wolf Horrell, John D'Orazio

**Affiliations:** 1 The Markey Cancer Center and the Department of Physiology, University of Kentucky College of Medicine, Lexington, KY 40536, USA; 2 The Markey Cancer Center and the Department of Pediatrics, University of Kentucky College of Medicine, Lexington, KY 40536, USA

**Keywords:** beta-defensin, UV radiation, qRTPCR, mRNA

## Abstract

In order to determine the effect of UV radiation on β-defensin 3 (BD3) expression in human skin, freshly-isolated UV-naïve skin was obtained from newborn male infants undergoing planned circumcision.  Skin explants sustained ex vivo dermis side down on RPMI media were exposed to 0.5 kJ/m ^2^ UVB, and biopsies were taken from the explant through 72 hours after radiation.  mRNA expression was measured by qRTPCR and normalized to TATA-binding protein.  BD3 expression at each time point was compared with an untreated control taken at time 0 within each skin sample.  Extensive variability in both the timing and magnitude of BD3 induction across individuals was noted and was not predicted by skin pigment phenotype, suggesting that BD3 induction was not influenced by epidermal melanization.  However, a mock-irradiated time course demonstrated UV-independent BD3 mRNA increases across multiple donors which was not further augmented by treatment with UV radiation, suggesting that factors other than UV damage promoted increased BD3 expression in the skin explants.  We conclude that BD3 expression is induced in a UV-independent manner in human skin explants processed and maintained in standard culture conditions, and that neonatal skin explants are an inappropriate model with which to study the effects of UV on BD3 induction in whole human skin.

## Introduction

The melanocortin 1 receptor (MC1R) is a G _s_-protein-coupled receptor expressed on melanocytes that regulates several key aspects of cutaneous UV responses. When bound by agonistic ligands, most notably α-melanocyte stimulating hormone (αMSH) ^1^, MC1R initiates a cascade of UV-protective events mediated by activation of adenylyl cyclase and generation of cAMP that result in melanin production and enhanced genome stability via enhancement of DNA repair ^2^. In addition to αMSH, MC1R signaling is regulated by other soluble ligands, most notably agouti signaling protein (ASIP) which antagonizes MC1R signaling, decreases cAMP levels, and diminishes downstream melanocyte responses such as pigment induction ^3, 4^. Recently, it has become clear that β-defensin 3 (BD3), known for its role in innate antimicrobial immunity, binds and influences MC1R signaling as a neutral MC1R agonist that blunts αMSH-mediated MC1R activation as well as ASIP-mediated MC1R antagonism ^5– 8^. Thus, BD3 may be an important regulator of MC1R-dependent melanocyte responses in the skin.

Because UV radiation is a major causative agent for melanoma and other skin cancers and because MC1R signaling mediates critical UV-protective responses such as melanization of the skin and melanocytic resistance to UV mutagenesis, it is important to understand how UV affects expression of MC1R ligands in the skin. αMSH levels increase in response to UV exposure of the skin. Cui and coworkers reported that UV promoted transcriptional increases in pro-opiomelanocortin (POMC), the protein precursor for αMSH, in a cell damage and p53-dependent manner in epidermal keratinocytes ^9^, supporting the hypothesis that melanocytic MC1R responses are modified by intracutaneous UV-regulated mechanisms. Similarly, recent studies reported that UVB radiation caused an increase BD3 mRNA and protein levels both *in vivo* and *in vitro* ^10^, either in a cell-autonomous, damage-dependent manner or in response to inflammatory mediators such as interleukin-1 (IL-1β) and tumor necrosis factor-α (TNFα) known to promote its induction ^11, 12^. In an effort to understand the mechanisms of how BD3 production may be influenced by UV radiation, we determined its expression in freshly isolated human skin explants. Here we report that BD3 expression increases in a UV-independent manner in neonatal human skin explants in response to processing and culturing of tissues *ex vivo*.

## Methods

*Neonatal foreskin explants.* Freshly-isolated, de-identified neonatal foreskins were collected from normal newborn infants undergoing planned circumcision from the University of Kentucky Birthing Center under an IRB-exempted protocol. Foreskins were collected only from patients who were consented prior to delivery. Samples were placed into 30 ml of Roswell Park Memorial Institute (RPMI) media (Life Technologies) and stored at room temperature for a maximum of four hours before processing. Samples were rinsed in phosphate buffered saline (PBS) + 1% penicillin streptomycin (Life Technologies), and dermal fat was manually removed by forceps to the point that explants would lie completely flat. Explants were placed in 3 cm cell culture dishes and floated dermal side down on 3 mL of RPMI media with 10% fetal bovine serum for 16–18 hours at 4°C until use. Prior to UV treatment, explants were divided into roughly equal-sized pieces. Following UV treatment, explants were maintained in 3 mL of RPMI + 10% fetal bovine serum + 1% penicillin streptomycin in a humidified incubator at 37°C with 5% CO _2_. The media was changed every 48 hours.

*Skin color measurement.* Skin reflective colorimetry was assessed with a CR-400 Colorimeter (Minolta Corporation, Japan) calibrated against a white background. Degree of melanization (darkness) was quantified as the colorimetric measurement on the *L axis (white-black axis) of the CIE standard color axis ^13^. The degree of pigmentation was determined by three independent measurements for each sample.

*UV exposure.* Skin explants were exposed (epidermal side up) to an overhead double bank of UVB lamps (UV Products, Upland, CA) to receive 0.5 kJ/m ^2^ UVB, a dose similar to that reported previously with respect to cutaneous BD3 induction *in vivo* ^10, 14^. UV emittance was measured with a Model IL1400A handheld flash measurement photometer (International Light, Newburyport, MA) equipped with separate UVB (measuring wavelengths from 265–332 nm; peak response at 290 nm) and UVA (measuring wavelengths from 315–390 nm; peak response at 355 nm) filters corresponding to International Light product numbers TD# 26532 and TD# 27108 respectively. Spectral output of the lamps was determined to be roughly 75% UV-B and 25% UV-A.

*Hematoxylin and eosin tissue staining*. Four neonatal skin explants were divided into two biopsies. One biopsy was untreated and harvested at time 0. The other was exposed to 0.5 kJ/m ^2^ UVB radiation and harvested at 24 hours. The biopsies were placed in 4% paraformaldehyde for 48 hours to fix the sample and subsequently placed in 70% ethanol. Samples were processed and stained for hematoxylin and eosin by the University of Kentucky Markey Biospecimen and Tissue Procurement Shared Resource Facility.

*mRNA isolation.* Total RNA was harvested from skin using TRIzol (Invitrogen). 25 mg of sample were placed in 500 ul of TRIzol and ground to a fine consistency using a dounce homogenizer. Homogenized sample was incubated for five minutes at room temperature. 100 uL of chloroform were added to each sample, and each sample was shaken vigorously for 15 seconds. Samples were incubated for 2–3 minutes at room temperature. Samples were centrifuged at 12,000 × *g* for 15 minutes at 4°C. RNA was isolated in the aqueous phase. RNA was precipitated with 250 uL of isopropanol. Sample was incubated at room temperature for 10 minutes and then centrifuged at 12,000 × *g* for 10 minutes at 4°C. Supernatant was removed. The RNA pellet was washed with 500 uL of ethanol and centrifuged at 7,500 × g for 5 minutes at 4°C. Supernatant was removed and the RNA pellet was dissolved in RNase free water. cDNA was reverse transcribed in a Mastercycler epgradient thermocycler (Eppendorf International) utilizing random hexamers and M-MLV reverse transcriptase (Promega).

*qPCR.* Quantitative real-time PCR (qRTPCR) analysis was performed using an Applied Biosystems 7500 Real Time PCR System (Life Technologies) (10 ng cDNA/reaction) utilizing TATA-binding protein (TBP) as a reference gene. Primer sets for TBP were 5´-CAGCGTGACTGTGAGTTGCT (left) and 5´-TGGTTCATGGGGAAAAACAT (right), for BD3 were 5´-TAGGGAGCTCTGCCTTACCA (left) and 5´-CACGCTGAGACTGGATGAAA (right), for TNFα were 5´-TCCTTCAGACACCCTCAACC (left) and 5´-AGGCCCCAGTTTGAATTCTT (right), and for tyrosinase were 5´-TACGGCGTAATCCTGGAAAC (left) and 5´-ATTGTGCATGCTGCTTTGAG (right) (Integrated DNA Technologies).

*Statistics and data analysis.* Correlation and linear regression analysis were performed using GraphPad Prism 5.0 (GraphPad Software, CA). Data were considered statistically significant if *p* values were less than 0.05 from multiple independent experiments.

## Results

**Figure 1.**
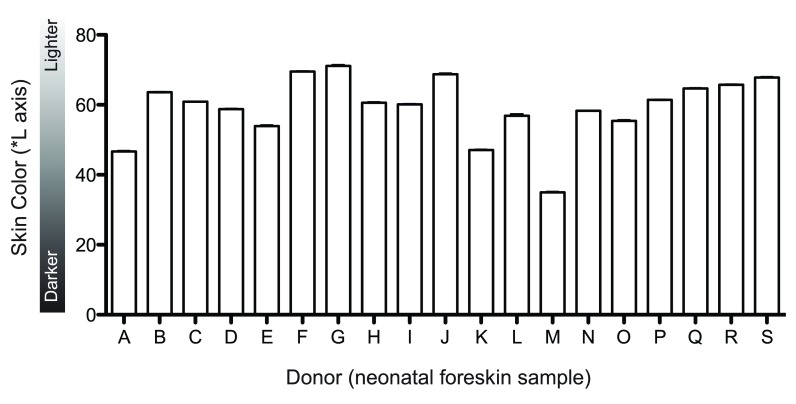
Degree of skin pigmentation from each donor. Skin color determination is shown for each sample. *L Score is measured by reflective colorimetry and represents color of the skin on a black-white axis. Lower *L score is indicative of a more darkly pigmented phenotype. Data represent the average *L score ± SEM for three measurements per skin sample.

**Figure 2.**
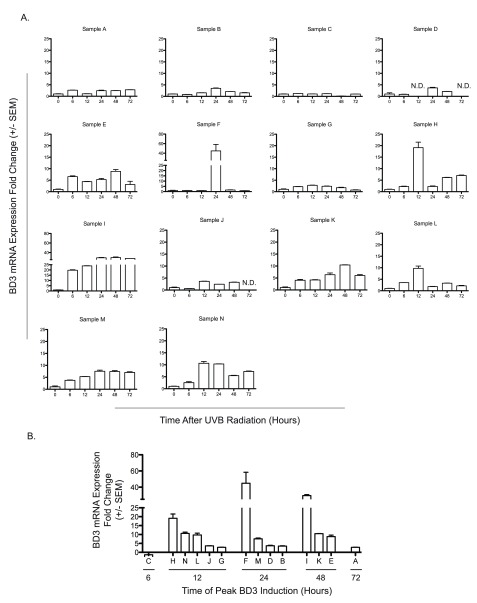
BD3 mRNA induction varies between individuals. **A**) Fourteen independent human skin explants (Samples A–N) were treated *ex vivo* with 0.5 kJ/m ^2^ UVB radiation. BD3 mRNA expression was determined at 6, 12, 24, 48, and 72 hours following UV treatment and compared to untreated matched controls. **B**) Time of maximal BD3 expression after UV radiation across samples. Peak BD3 mRNA expression for human skin explants (n=14) is arranged by time of maximal induction for each individual donor. qRTPCR was performed in duplicate for each sample, and results are expressed as mean fold change over control ± SEM.

**Figure 3.**
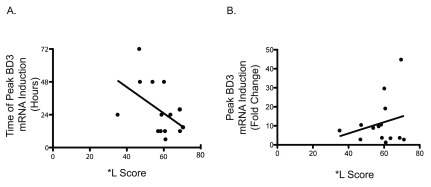
Relationship between donor skin color and BD3 expression. **A**) *L score versus peak BD3 mRNA induction. qRTPCR was performed in duplicate for each sample; data represent mean BD3 induction for 14 human skin explants. There was no correlation between donor *L score and amplitude of BD3 induction (r ^2^ = 0.057, p = 0.41). **B**) *L score versus time of peak BD3 mRNA induction. qRTPCR was performed in duplicate for each sample, and data represent mean BD3 induction for 14 human skin explants. Although a weak negative trend existed between donor *L score and time of BD3 induction, the correlation was not statistically significant (r ^2^ = 0.234, p = 0.08).

To understand the effects of UV radiation on BD3 expression in human skin, freshly-isolated foreskins were exposed to 0.5 kJ/m ^2^ UVB. Fourteen de-identified samples were obtained from normal healthy male infants undergoing elective circumcision before discharge from the neonatal nursery. Skin pigmentation was measured for each sample three independent times by reflective colorimetry in order to estimate melanin content of the epidermis. The skin samples exhibited a range of melanization as determined by the *L score which quantifies color on a black-white color axis (a lower *L score is indicative of a blacker/darker color and correlates with epidermal eumelanin content ^15^). The majority of the samples were derived from light-skinned infants, however at least 3 samples were darker in color ( Figure 1). Skin explants were exposed to 0.5 kJ/m ^2^ UVB, and biopsies were taken from the explants at 6, 12, 24, 48, and 72 hours following UV exposure. BD3 mRNA expression was measured by qRTPCR at 6, 12, 24, 48 and 72 hours after radiation, normalized to TBP, and compared to an unirradiated control taken at time 0. Due to the small size of the skin explants (roughly 1 cm ^2^), it was not possible to have a time-matched mock-irradiated control at each time point, therefore values were normalized to unirradiated controls from each skin sample. We noted extensive variability in both the timing and magnitude of BD3 induction across individuals ( Figure 2A). Normalized BD3 fold induction ranged from 1.3-fold to 44.8-fold, and peak induction ranged from 6–72 hours depending on the sample ( Figure 2B). We tested whether the amount of BD3 expression correlated with skin pigmentation, hypothesizing that more melanin in the skin might inhibit UV penetration into the skin and therefore blunt UV effects on BD3 expression. In fact, BD3 expression did not appear to be influenced by pigment phenotype, as manifested by a positive trend between higher BD3 expression and darker skin samples ( Figure 3A; r ^2^ = 0.057, p = 0.41). Similarly, a negative trend between skin color and time of peak BD3 expression was observed, although this too did not reach statistical significance ( Figure 3B; r ^2^ = 0.234, p = 0.08).

**Figure 4.**
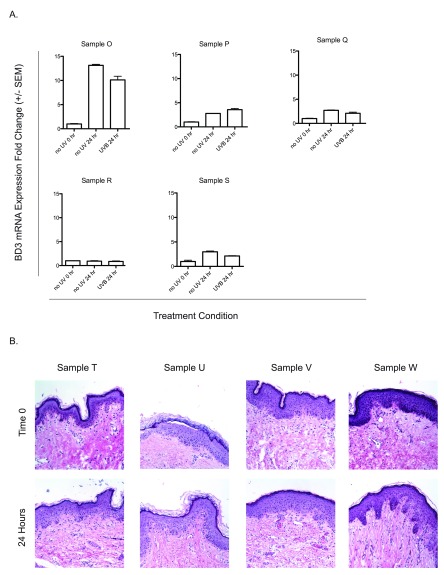
UV-independent BD3 expression in human skin explants cultured *ex vivo*. **A**) UVB independent induction of BD3. Five human neonatal skin explants (Samples O–S) were treated *ex vivo* with 0.5 kJ/m ^2^ UVB radiation. BD3 mRNA expression of UV-treated samples and unirradiated time-matched controls were compared to unirradiated time-matched controls taken at time 0. qRTPCR was performed in duplicate for each sample, and data represent the mean fold change over the untreated control taken at the time of UV treatment ± SEM. **B**) Histological analysis of neonatal skin samples at time 0 or 24 hours after UVB irradiation (0.5 kJ/m ^2^; Samples T–W). Tissues were stained with hematoxylin and eosin to assess tissue degradation.

We then considered the possibility that BD3 expression might be affected simply by time in culture and measured BD3 expression over time in samples exposed to 0 or 0.5 kJ/m ^2^ UVB exposure. Each of five explants were divided into three sections and sampled either at time 0 (no UV) or at 24 hours following exposure to either 0 or 0.5 kJ/m ^2^ UVB. Similar to prior experiments, BD3 expression was measured by qRTPCR and normalized to TBP, however values could also be compared with mock-irradiated, time-matched conditions. We observed clear induction of BD3 expression in each of the mock-irradiated samples over time ( Figure 4A), and exposure to 0.5 kJ/m ^2^ UVB did not substantially alter BD3 mRNA expression when compared to individual mock-irradiated time-matched controls. We assessed whether the processing of the samples led to sample degradation via immunohistochemistry. Staining revealed that after 24 hours of *ex vivo* treatment, the samples appeared similar to those at time 0 and suggested their viability ( Figure 4B). These data suggest that either tissue removal or the process of culturing skin explants *ex vivo* in our culture conditions is sufficient to enhance BD3 expression in whole human neonatal skin and that the addition of 0.5 kJ/m ^2^ UVB does not impact BD3 expression in this setting.

**Figure 5.**
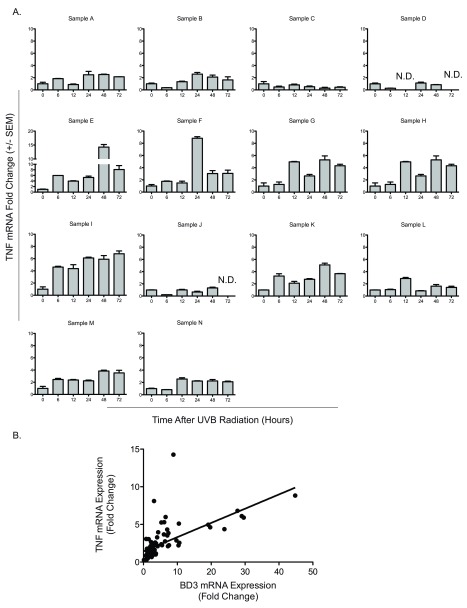
TNFα mRNA induction in human skin explants cultured *ex vivo*. **A**) TNFα expression over time among 14 distinct donors after UV radiation. Fourteen independent neonatal human skin explants (Samples A–N) were treated *ex vivo* with 0.5 kJ/m ^2^ UVB radiation. TNFα mRNA expression was determined at 6, 12, 24, 48, and 72 hours following UV treatment and compared to matched untreated controls. **B**) Correlation of BD3 and TNFα mRNA expression over time. BD3 and TNFα mRNA expression were compared among fourteen human skin explants (Samples A–N) at 0, 6, 12, 24, 48, and 72 hours. BD3 and TNFα mRNA expression correlated over time (r ^2^ = 0.335, p<0.0001). qRTPCR was performed in duplicate for each sample, and results are expressed as mean fold change over control ± SEM.

**Figure 6.**
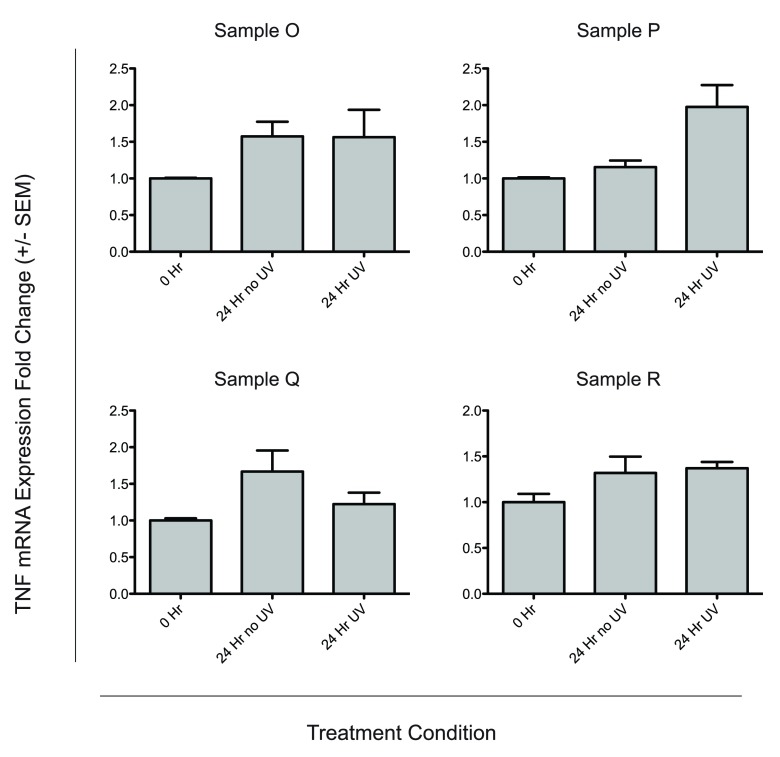
UV-independent TNFα expression in human skin explants cultured *ex vivo*. **A**) UVB independent induction of TNFα. Four neonatal human skin explants (Samples O–R) were treated *ex vivo* with 0.5 kJ/m ^2^ UVB radiation. TNFα mRNA expression for UV-treated biopsies and unirradiated time-matched controls were compared to unirradiated tissue-matched controls taken at time 0. qRTPCR was performed in duplicate for each sample, and data represent the mean fold change over the untreated control taken at the time of UV treatment ± SEM.

Because cytokines, particularly TNFα are known to regulate BD3 expression, we tested whether TNFα gene expression was induced in the human neonatal skin samples following UV radiation. TNFα mRNA levels were assessed via qRTPCR at 6, 12, 24, 48, and 72 hours following UVB radiation, normalized to TBP, and compared to unirradiated controls. TNFα mRNA levels increased with time after UV in the majority of samples tested ( Figure 5A). Normalized TNFα mRNA induction ranged from 0–14.3 fold across samples. TNFα and BD3 induction weakly correlated over time ( Figure 5B, r ^2^ = 0.335, p<0.0001) suggesting a relationship between the two genes. UV-independent TNFα induction was then assessed in four additional samples. We observed that in three of four samples, TNFα expression increased in culture without UV ( Figure 6), suggesting that tissue processing may increase TNFα levels independently from UV.

**Figure 7.**
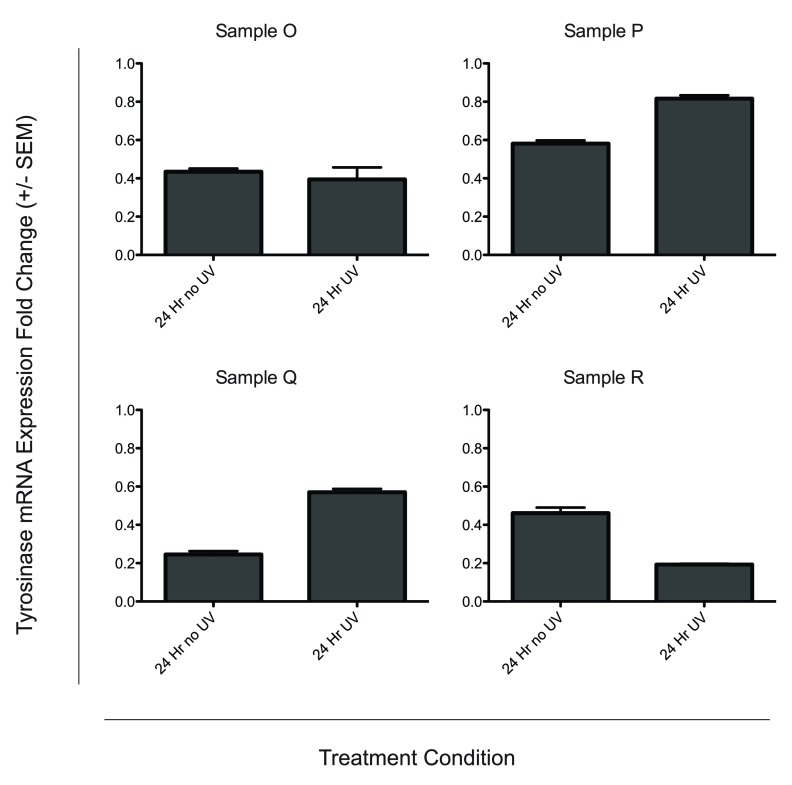
Tyrosinase mRNA expression in human skin explants cultured *ex vivo*. Four neonatal human skin explants (Samples O–R) were treated *ex vivo* with 0.5 kJ/m ^2^ UVB radiation. Tyrosinase mRNA expression was determined for UV treated biopsies and unirradiated time-matched controls and compared to tissue-matched unirradiated controls taken at time 0. qRTPCR was performed in duplicate for each sample, and data represent the mean fold change over the untreated control taken at the time of UV treatment ± SEM.

We then assessed whether *ex vivo* culture conditions used in these experiments affected other genes known to be regulated following UV radiation. Tyrosinase gene expression was measured in four human neonatal skin samples 24 hours after mock- or UV-irradiation. UV increased levels of tyrosinase gene expression in two of the four samples ( Figure 7), suggesting that these culture conditions may be appropriate for other genes if properly controlled.

Update 1: Colorimetry measurements from each donor Skin pigmentation was determined via reflective colorimetry and is represented by an *L score. The *L score was measured three times for each sample. The “A” column represents each donor. The “B”, “C”, and “D” columns represent the first, second, and third measured *L score respectively.

Update 2: Cycle threshold values for qRTPCR Cycle threshold (C _T_) values were calculated for BD3, TNFα, tyrosinase, and TBP (housekeeping genes) for each donor. C _T_ values were determined in duplicate for each sample. The “A” column represents the donor. The “B” column represents the sample treatment for the donor. The “C” column represents the target mRNA measured. The “D” column represents the C _T_ value determined for that sample.

## Conclusions/discussions

In an effort to develop a model in which to study UV induction of cutaneous BD3, we measured its expression over time in UV-naïve human skin explants. Although there was a high degree of variability in the magnitude and kinetics of BD3 induction between samples harvested from different donors, we observed BD3 up-regulation in each case. To control for the possibility that tissue processing and/or *ex vivo* culture conditions might impact BD3 expression in the explants, we compared BD3 mRNA levels between mock-irradiated versus UV-treated sections of skin samples harvested from the same donor. This experiment, which included samples from five donors, indicated that BD3 expression increased over time irrespective of UV exposure (at 0.5 kJ/m ^2^), suggesting that BD3 expression is induced in human skin explants in a UV-independent manner.

BD3 expression has been reported to be up-regulated in wound healing processes ^16^, therefore it might be plausible that its increase over time in skin explants may be related to normal wound physiologic processes activated by surgical excision of the skin and/or its processing after harvest. The small size of the skin samples isolated from neonatal circumcision (on average 1–1.5 cm ^2^) implies that the majority of the tissue in the explant will be in close proximity to at least one cut surface, raising the possibility of local trauma-induced factors contributing to BD3 expression in the samples. TNFα is an inflammatory cytokine known to be upregulated in the wound healing process, and TNFα mRNA was also induced in the skin samples independently from UV radiation. TNFα induction over time correlated with BD3 mRNA induction providing further support that BD3 induction in the skin explants may be related to normal wound healing processes.

Our data do not rule out the possibility that the wounding response following surgical excision and processing may be sufficiently robust as to prevent further induction by UV. Tyrosinase mRNA levels, however, were induced following UV radiation in 50% of the samples suggesting some genes regulated by UV can be induced in our *ex vivo* model. Alternatively, it is possible that one or more factors involved in sustaining the skins in culture (media, temperature, oxygen tension, pH, etc.) may have promoted BD3 expression in the explants. We do not as yet understand the mechanism(s) underlying variability of BD3 induction amplitude or kinetics observed between samples, however it is possible that wounding or inflammatory responses induced by tissue removal may vary between normal individuals.

Previous studies have utilized adult human skin explants and reported an induction of BD3 mRNA following UV radiation in *ex vivo* culture conditions ^10^. It is possible that neonatal skin explants behave differently than adult skin explants, accounting for the inconsistent results between the two studies. In general, neonatal immune responses are less mature than those of adults, perhaps contributing to these observations. In addition, prior UV exposures of adult-derived skin tissues may not be controlled as are skin explants from UV-naïve neonatal foreskins which may also impact results. We conclude that because of confounding variables involved in their generation and maintenance, neonatal foreskin explants processed via the conditions outlined above may not be an appropriate model to isolate the effects of UV on BD3 expression in the skin, however other models may still be appropriate.

## Consent

De-identified neonatal foreskin samples were obtained from the University of Kentucky’s Chandler Medical Center Newborn Nursery without accompanying clinical information under an institutionally-reviewed IRB-exempted status.

## Data availability

F1000Research: Dataset 1. Update 1: **Colorimetry measurements from each donor**, 10.5256/f1000research.5794.d43456 ^17^

F1000Research: Dataset 2. Update 1: **Cycle threshold values for qRTPCR**, 10.5256/f1000research.5794.d43457 ^18^
