# Supplementary material for: UV-independent induction of beta defensin 3 in neonatal human skin explants
Source: F1000Res. 2015 Feb 19;3:288. Originally published 2014 Nov 21. [Version 2] doi: 10.12688/f1000research.5794.2 (PMC4304222; doi:10.12688/f1000research.5794.2)
Supplement: Peer review history

# Peer review: Referee response for version 1

**DOI:** 10.5256/f1000research.6194.r7161

**Published:** 2015-01-20

**Authors:** Pamela Cassidy

**Affiliations:** 1 Department of Dermatology, Oregon Health Sciences University, Portland, OR, USA

The authors describe their studies of the effects of UV-irradiation on the expression of beta defensin on human neonatal foreskins treated and cultured *in vivo*. After finding variable responses between donors which did not correlate with skin phototype, they investigated the possibility that beta defensin expression was affected by culture conditions. They found that there were no differences between mock- and UV-irradiated samples. We have seen similar changes in genes of interest in the mock-treated tissues using *ex vivo* skin cultures. This study is valuable in that it highlights the need to include proper controls in this system. I have several additional minor comments to make about the manuscript and data:

1. The protocol for mRNA isolation notes that tissue is ground in trizol and RNA is purified from the aqueous layer. There will not be two layers until chloroform is added and this should be included in the protocol.
2. Figure 1 claims to represent degree of skin pigmentation +/- SEM but there are no error bars.
3. The qPCR data shows Ct values as high as 32.9 for beta defensin, with almost all of the time=0 samples above 29. Many qPCR assays are not linear in this range. Did the authors do an examination of the performance of their primers in this range? Although these high Ct values make me a little skeptical about the absolute values of the fold change reported in figures 2 and 5, I am nevertheless persuaded that the differences between mock- and UV-treated samples is negligible as the authors conclude.

I have read this submission. I believe that I have an appropriate level of expertise to confirm that it is of an acceptable scientific standard.

# Response

**Published:** 2015-02-12

**Authors:** John D'Orazio

**Affiliations:** University of Kentucky, USA

We sincerely thank you for your thorough reading and insightful comments. We have provided a point-by-point response to each of your concerns.

- Methods: The protocol for mRNA isolation via Trizol is not complete.
  - We have expanded our methods to include these details.
- General Concern: Figure 1 claims to represent degrees of skin pigmentation +/- SEM but there are no error bars.
  - We have expanded our methods to be more specific with how the measurements were determined. The degree of pigmentation for each skin sample was determined three times by colorimetry measurement. Neonatal skin samples were, as a rule, fairly uniform in their pigmentation. Their homogeneity between measurements therefore resulted in very small standard error of the mean values.

# Peer review: Referee response for version 1

**DOI:** 10.5256/f1000research.6194.r6760

**Published:** 2014-12-23

**Authors:** Lidia Kos

**Affiliations:** 1 Department of Biological Sciences, Florida International University, Miami, FL, USA

The authors attempt to establish a skin explant model to investigate the effect of UV exposure on the expression levels of b-defensin 3, an antimicrobial peptide that also binds to Melanocortin 1 receptor and attenuates downstream signaling. They find extensive variability in the expression levels of b-defensin 3 in the UV-induced samples and cannot demonstrate that is in in fact up-regulated. They do show some minor increase in b-defensin 3 expression over time that is independent of UV irradiation and suggest that this may be due to a wound healing response to the skin excision process. The authors should have included in their analysis a few more genes known to be upregulated after UV exposure (for example, MC1R) both in keratinocytes and melanocytes. This way, they would have been able to define whether their culture system was improper to study b-defensin3 regulation specifically. In the mock irradiated experiment they should have also looked at levels of IL1 and TNFalpha to support their hypothesis that b-defensin 3  increases as a result of a wound healing response. In the paper by Glaser *et al (*2009) where b-defensin 3 was reported to be up-regulated after UV exposure, the authors used explants of adult skin. Is it possible that newborn and adult skin (perhaps based on the anatomy or level of cellular differentiation) respond differently to UV exposure that could explain the disparate results?

I have read this submission. I believe that I have an appropriate level of expertise to confirm that it is of an acceptable scientific standard, however I have significant reservations, as outlined above.

# Response

**Published:** 2015-02-12

**Authors:** John D'Orazio

**Affiliations:** University of Kentucky, USA

We sincerely thank you for your thorough reading and insightful comments. We have provided a point-by-point response to each of your concerns.

- General comment: The authors should have included a few more genes known to upregulated after UV exposure in both keratinocytes and melanocytes.
  - We have included the induction of additional genes including tyrosinase in four human neonatal skin samples. We determined that tyrosinase gene expression was induced following UV radiation in half of the skin samples suggesting the model with our culture conditions may be appropriate to study other genes.
- General Comment: The authors should have looked at levels of IL1 and TNF alpha to support their hypothesis that BD3 increases as a result of wound healing.
  - We have included the gene expression data for TNF alpha following UV radiation and determined that its expression correlates with BD3 expression.
- General Comment: Is it possible that the neonatal skin explants behave differently than the adult skin explants?
  - Yes, this is a possibility, however as our study was limited to neonatal explants, we cannot directly address it with data.  However, we thank the reviewer for this comment and have raised this caveat in our revised discussion.

# Peer review: Referee response for version 1

**DOI:** 10.5256/f1000research.6194.r6759

**Published:** 2014-12-04

**Authors:** Zalfa Abdel-Malek

**Affiliations:** 1 Cancer Institute, University of Cincinnati, Cincinnati, OH, USA

The study by Horell and D'Orazio aimed at addressing the production of BD3, a MC1R antagonist, in neonatal human foreskins, using skin explants, and its possible regulation by UV irradiation. The authors did not find a correlation between the pigmentary status of the skin and BD3 production, or a consistent effect of UV radiation. However, they noted altered production of BD3 over time in the skin explants, which they attributed to a wound repair-like reaction, or to inadequate culture conditions for the skin explants. They concluded that the use of foreskins in organotypic culture is not an appropriate model to assess BD3 production.

It is not clear from the report exactly how the skin explants were maintained for the duration of the experiments. Were they maintained in a humidified incubator at 37°C? Was the culture medium changed daily? Obviously, the culture conditions can affect the viability of the explants. Did the authors check on the viability of the skin, especially at the end of the experiment (e.g. by examining the histology after H&E staining)? This is important, and the "health" of the skin can explain the erratic production of BD3. If the viability of the skin is compromised, this will have a generalized effect on its metabolic state.

Since IL-1 is known to stimulate BD3 expression, did the authors check the levels of IL-1, particularly after UV exposure?

If the change in BD3 production is due to a wound healing-like response, the authors might have to consider cutting the foreskins into equal parts at the beginning of the experiment, to avoid taking biopsies at different time points.

It could very well be that the experimental conditions are responsible for the unexpected results that were obtained. So the conclusion that using foreskin explants is not an appropriate model might not be necessarily true.

I suggest that the authors submit this report as a "methodology" report, after investigating the concerns raised above.

I have read this submission. I believe that I have an appropriate level of expertise to confirm that it is of an acceptable scientific standard, however I have significant reservations, as outlined above.

# Response

**Published:** 2015-02-12

**Authors:** John D'Orazio

**Affiliations:** University of Kentucky, USA

We sincerely thank you for your thorough reading of our manuscript and insightful comments. We have provided a point-by-point response to each of your concerns.

- Methods: It is unclear as to how the skin explants were maintained.
  - We have expanded the methods to include these details.
- General Comment: Did the authors check the viability of skin samples?
  - We have included hematoxylin and eosin stained histological slides for the skin explants at 0 and 24 hours following culture conditions to assess the viability of the samples and have determined the samples were still viable. Furthermore induction of TNF alpha suggests viability of the tissues over the time course of the experiment.
- General Comment: Did the authors check the level of IL-1B following UV radiation?
  - We have determined the levels of TNF alpha which is also known to induce beta-defensin 3 expression. TNF induction appears to correlate with the beta-defensin 3 induction suggesting they are related.
- Methods: The authors may consider cutting the foreskins into equal parts at the beginning of the experiment to determine whether the change is due to a wound healing response.
  - The samples were divided into equal parts at the beginning of the experiment prior to UV radiation. We apologize for the confusion in the methods and have updated the methods to include this information.
- General Comment: The conclusion that the use of human neonatal foreskins may not be an appropriate model to study BD3 induction may not be true and the results may be due to the experimental conditions. 
  - We thank the reviewer for this comment and have adjusted our discussion to accommodate it.
- General Comment: The authors should submit this report as a “methodology report.”
  - Given the fact that our data raise important caveats about the use of neonatal human foreskins to study BD3 induction, we feel that our findings may be better reported as a research article rather than as a methodology report. 
